# Uncertainties in the Shuttle Radar Topography Mission (SRTM) Heights: Insights from the Indian Himalaya and Peninsula

**DOI:** 10.1038/srep41672

**Published:** 2017-02-08

**Authors:** Manas Mukul, Vinee Srivastava, Sridevi Jade, Malay Mukul

**Affiliations:** 1 KIIT University, Bhubaneswar, 751024 India; 2 Continental Deformation Laboratory, Dept of Earth Sciences, Indian Institute of Technology Bombay, Mumbai, 400076 India; 3 CSIR-4PI, CSIR Fourth Paradigm Institute (Formerly CSIR-CMMACS), Wind Tunnel Road, Bangalore, 560037 India

## Abstract

The Shuttle Radar Topography Mission (SRTM) Digital Terrain Elevation Data (DTED) are used with the consensus view that it has a minimum vertical accuracy of 16 m absolute error at 90% confidence (Root Mean Square Error (RMSE) of 9.73 m) world-wide. However, vertical accuracy of the data decreases with increase in slope and elevation due to presence of large outliers and voids. Therefore, studies using SRTM data “as is”, especially in regions like the Himalaya, are not statistically meaningful. New data from ~200 high-precision static Global Position System (GPS) Independent Check Points (ICPs) in the Himalaya and Peninsular India indicate that only 1-arc X-Band data are usable “as is” in the Himalaya as it has height accuracy of 9.18 m (RMSE). In contrast, recently released (2014–2015) “as-is” 1-arc and widely used 3-arc C-Band data have a height accuracy of RMSE 23.53 m and 47.24 m and need to be corrected before use. Outlier and void filtering improves the height accuracy to RMSE 8 m, 10.14 m, 14.38 m for 1-arc X and C-Band and 3-arc C-Band data respectively. Our study indicates that the C-Band 90 m and 30 m DEMs are well-aligned and without any significant horizontal offset implying that area and length computations using both the datasets have identical values.

Digital Terrain Elevation Data (DTED) are being used extensively world-wide. High-quality DTED from the Shuttle Radar Topography Mission (SRTM)^1^, acquired using C- and X-Band antennae with swath widths 225 and 45 km and wavelengths 5.6 and 3 cm respectively, is a very popular source of digital topography data for its widespread coverage of about 80% of the earth’s surface between 60°N and 56°S latitudes. The SRTM data collected by the U.S. National Aeronautics and Space Administration (NASA), the German Aerospace Center (DLR) and the Italian Space Agency (Agenzia Spaziale Italiana; ASI) in February 2000^2^,^3^,^4^ are available at 1-arc or 30 m horizontal resolution in C-Band (C30) and X-Band (X30) worldwide since August, 2015. The C-Band data are more extensive due to the greater swath of the antennae^5^ and C-Band 3-arc data with 90 m horizontal resolution (C90) data have been extensively used for the last 15 years. The vertical resolution of these data, however, is variable and needs to be carefully and systematically studied.

The initial validation of global C-Band 90 m (C90) SRTM heights used dual-frequency Real Time Kinematic (RTK) Global Positioning System (GPS)^6^,^7^. GPS Independent Check Points (ICPs) of various precisions have been used for the accuracy assessment of SRTM heights. For example, the International Global Navigation Satellite System Service (IGS) Network^8^ was used to assess the accuracy of C90 SRTM heights. Regional validations of C90 SRTM heights were also carried out in Poland^9^,^10^, India^11^, Greece^12^, Thailand and US^13^ using GPS based ICPs. These studies indicate that the accuracy of SRTM heights decreases with increasing topographic slope and elevation. For example, in Poland the percentages of SRTM data having vertical absolute error below 16 m were 82% (C90) and 74% (X30) and the high error was attributed to high slope angles and initial void distribution^10^. Similarly, in India a Root Mean Square Error (RMSE) of 3.55 m, 11.44 m and 19.64 m for plains (elevation variation ~40 m), moderately (elevation variation ~300 m) and highly undulating (elevation variation ~4000 m) Himalayan topography respectively^11^ implies that the SRTM goal was achieved only in the plains. The Himalaya is also known to contain the greatest concentration of SRTM data voids with interpolated data^12^ that impact the accuracy of SRTM heights^10^. Moreover, there is widespread use of SRTM data in various earth-science disciplines particularly in and around the Himalaya^14^,^15^,^16^,^17^,^18^ making it important to assess the uncertainty associated with SRTM C- and X-Band heights in the Himalaya as a high variability in SRTM height errors are observed here^11^.

The SRTM targeted a vertical accuracy of 16 m absolute error at 90% confidence or Linear Error 90 (LE90)^2^,^3^,^19^,^20^ that is equivalent to RMSE of 9.73 m (LE90 = 1.6449*RMSE^21^). Initial worldwide validation of the C-Band 90 m SRTM (C90) heights demonstrated that this goal had been achieved^6^,^7^. Therefore, the minimum vertical accuracy of the global SRTM data, although quite variable, was accepted to be 16 m (LE90)/~10 m RMSE^6^,^7^. The LE90 for Africa, Australia, Eurasia, Islands, N. America, S. America were reported as 5.6 m, 6.0 m, 6.2 m, 8.0 m, 9.0 m, and 6.2 m with equivalent RMSE of 3.4 m, 3.7 m, 3.8 m, 4.9 m, 5.5 m and 3.8 m respectively^6^,^7^,^8^. Thereafter, SRTM data is being routinely used with the consensus view that it possesses 90 m or 30 m horizontal resolution and a vertical LE90 accuracy ranging between 5–9 m and less than 16 m. This, however, is misleading because (1) Datasets used as Independent Check Points (ICPs) for the global validation of SRTM data were limited in their spatial distribution^6^,^7^, e. g. no ICPs existed in the Indian sub-continent. (2) Existing studies indicate that the accuracy of SRTM data decreases with the increase in slope and elevation^9^,^11^,^13^. (3) Large outliers and voids exist in the SRTM dataset^6^,^7^,^8^. This suggests that assessment of the local accuracy of SRTM heights needs to be carried out before its statistically meaningful use and a single representative value for entire continents is oversimplification. Nevertheless studies using “as is” SRTM data with no real mention of vertical uncertainties are widespread in earth science. For example, geomorphometric analysis^22^, vegetation cover studies^23^, analysis of avulsion threshold of rivers^24^, assessment of tsunami^25^, modeling of soil particle size distribution^26^, measuring the surface elevation changes of glaciers^27^, morphology of terraces^28^ and fault geometry of active folds^29^ have used SRTM data “as is” without outlier analysis or propagation of errors associated with vertical (height) uncertainties in SRTM data.

We address the following important issues related to the use of SRTM data and its vertical (height) uncertainty using ~200 high-precision GPS points as ICPs (Fig. 1, Supplementary Table S1) in India: (1) The minimum horizontal precision of GPS ICPs required for accurate pixel registration for 90 m, 30 m and 15 m resolution SRTM DTED for defining a quantitative threshold for selection of ICPs. (2) The accuracy of C-Band and X-Band SRTM data in India and the Himalaya. (3) Accuracy of the SRTM void-filling interpolations in the Himalaya. (4) An effective methodology for the use of SRTM data.

## Results

The results of the Shapiro-Wilk’s normality test (p > 0.05), inspection of the skewness and kurtosis measures and standard errors^30^,^31^,^32^ and their normal Q-Q plots (Fig. 2) show that the X-Band errors were approximately normally distributed (p = 0.862) with a skewness of 0.171, standard error (SE) of 0.304 and a kurtosis of −0.212 (SE = 0.599). Both the C90 and C30 errors fail the Shapiro-Wilk’s (p > 0.05) normality test with respective ‘p’ values of 0.008 and 0.002. C90 had a skewness of 0.479 (SE = 0.176) and kurtosis of 0.266 (SE = 0.350) where as the C30 skewness and kurtosis was 2.86 (SE = 0.175) and −0.06 (SE = 0.348) respectively. Having established that, we first address the issue of minimum precision of the position of the ICPs required for accurate pixel registration and then analyse the “as is”, void and outlier filtered SRTM data (Table 1) and study the effect of re-sampling on vertical accuracy of C-Band SRTM data by the analysis of outlier-filtered 191 C90, 193 C30 and 194 C30_15 ICPs (Table 2).

### Minimum horizontal precision of ICPs for accurate pixel registration

The elevation values for C-Band 90 m, 30 m and 15 m were extracted by registering pixels with ICPs whose positions were computed to 8 places after decimal. The latitude and longitude for each of the ICPs were taken starting from precision of 1 up to 8 places after decimal. Hence, 8 sets (P1, P2, P3, P4, P5, P6, P7, P8) of elevation data were obtained with the same set of ICPs by varying the precision of their latitude and longitude. For each of the 8 datasets obtained above, the RMSE of the difference between the ICP elevation and the pixel elevation was computed. The RMSE for 90 m resolution data did not change after the P5 dataset (Supplementary Table S2; Fig. 3). For 30 m resolution (and re-sampled 15 m) data the RMSE was constant after P6 (Supplementary Table S2; Fig. 3).

### Accuracy of complete C90, C30 and X30 “as is”, void removed and outlier filtered data

The C90, C30 and X30 “as is” datasets consisted of 220, 219 and 65 points with RMSE of 47.24, 23.53 and 9.18 m respectively (Table 1, Fig. 4). We found that 13 C90 and 11 C30 ICPs were located within the SRTM void region and the RMSE for C90 and C30 datasets after filtering these voids were 26.98 and 23.57 m respectively (Table 1, Fig. 4). There were no X30 data from the SRTM void region. The datasets were further filtered to remove the 16 C90, 15 C30 and 3X30 outliers (Supplementary Table S3) and their RMSE computed to 14.38, 10.14 and 8.0 m respectively (Table 1, Fig. 4).

### Accuracy of the SRTM void-filling interpolations in the Himalaya

A total of 13 ICPs were located in C-Band SRTM void regions at GPS elevations ranging between 1300–3600 m (Supplementary Table S4; Fig. S1). The SRTM values obtained at these ICPs have been generated by interpolation algorithms^33^,^34^ using measured SRTM data nearby to estimate elevation values in the voids. The ICPs located in the SRTM voids, therefore, provide an insight into the quality of the interpolation algorithms by comparison of the measured and interpolated values (Supplementary Table S4). The MAE and the RMSE of these 13 points for C90 were 121.63 and 161.78 m respectively. The C30 MAE and RMSE were significantly lower at 17.15 and 22.64 m. The lowest MAE and RMSE were 12.84 and 20.22 m respectively for the C30_15 re-sampled points (Supplementary Table S4).

### Accuracy of void and outlier-filtered C90, C30, re-sampled C-Band 30 m (C30_15) and X30 data in the Indian Himalaya and the Peninsula

We categorized the Himalayan ICPs into Himalayan foreland, foothills and higher Himalaya on the basis of their GPS heights. The Himalayan foreland consisted of 21 C90, 22 C30 and 7X30 ICPs below the elevation of 300 m. The RMSE for outlier and void filtered C90, C30 and X30 datasets were 9.96, 8.08 and 5.03 m respectively (Table 2). The Himalayan foothills consisted of 40 C90, 39 C30 and 9X30 ICPs having GPS elevations between 300 m to 2000 m. This outlier and void filtered dataset showed RMSE of 15.95, 10 and 5.40 m for C90, C30 and X30 datasets respectively (Table 2). The Higher Himalaya consisted of 30 C90, 32 C30 and 13X30 ICPs set up at elevations greater than 2000 m. The RMSE for outlier and void filtered C90, C30 and X30 data were 18.63 m, 13.71 and 7.40 m respectively (Table 2). In addition, we also analysed re-sampled C30_15 data from the outlier and void filtered C30 data. The RMSE of the C30_15 data in the Himalayan foreland, foothill and higher Himalayan regions were 7.58 m, 9.06 m and 13.37 m respectively (Table 2).

The Peninsular outlier and void filtered dataset consisted of 100 C90, 100 C30 and 32X30 stations spread across the Indian subcontinent south of the river Ganges with mean GPS elevation of ~258 m. The C90 and C30 RMSE for this dataset were 12.97 and 9.22 m respectively. The X30 RMSE for this dataset was 9.34 m. For the re-sampled C30_15 dataset, the RMSE was 9.26 m (Table 2).

## Discussion

We now address the issues we raised related to the use of SRTM data and their vertical (height) uncertainties using ~200 high-precision ICPs (Supplementary Table S1) in India.

How precise does the position of an ICP need to be? Our analysis indicates that an ICP position expressed in latitude and longitude degrees with a minimum precision of 5 places after decimal (P5 in Fig. 3) is required for accurate pixel registration for 90, 30 and 15 m resolution DEMs as the RMSE remains unchanged for higher precision ICP positions (Fig. 3; Supplementary Table S2). This seems true for ICPs used for analysis of “as is” SRTM as well as “re-sampled” C30_15 dataset. As 1 decimal degree gives a precision up to ~111 km at the equator, latitude and longitude measurements of 6 places after decimal approximate to about 0.11 m of precision. The dual-frequency differential static GPS data analysis used in this study gives ICP positions precise to 4 places of decimal with centimetre-level accuracy in the vertical component and to 8 places after decimal with millimetre-level accuracy in the horizontal component. In comparison, the dual-frequency Real Time Kinematic GPS gives a horizontal and vertical precision of 5 and 3 places after decimal respectively with decimetre-level accuracy in both as seen from our field observations. Hence, the type of GPS receiver used to measure the position of an ICP impacts the accuracy assessment and a horizontal precision lower than 5 places results in inaccurate pixel registration of the ICPs. The accuracy of the C90 and C30 DEMs also depends on how the DEMs are aligned with each other^35^. Any misalignment or horizontal offset of the DEMs will introduce elevation differences that increase with steeper slopes^35^. Hence, detection and correction of horizontal offset, if any, between the C90 and C30 DEMs is essential before assessing their accuracy. The detection of horizontal offset is done by comparing the elevation difference raster with the hillshade of the terrain which is a function based on aspect and terrain slope^35^. The C-Band 90 m and 30 m DEMs are aligned without any horizontal offset as the elevation difference between the two DEMs do not show significant similarity with the hillshade (Fig. S2). Also a strong correlation of 0.99999 exists between the two DEMs indicating a high dependency between the pixel values of the two DEMs. This also implies that area and length computations using C-Band 90 m and 30 m DEMs would have near identical values^36^.

C-Band SRTM data in the Himalaya contain data voids and we found 13 ICPs (Fig. S1) located within voids. However, none of these ICPs contained X30 data. The MAE and the RMSE of these 13 points for “as is” C90 was 121.63 and 161.78 m respectively but decreased to 17.15 and 22.64 m for C30 data indicating that void-filling interpolation algorithms are much better for C30 data. The C30_15 data, re-sampled from “as is” C30 data, had MAE and RMSE of 12.84 and 20.22 m suggesting that re-sampling of C30 marginally improved the height errors in the SRTM voids (Supplementary Table S4). As the SRTM voids occur at high elevations (1300–3600 m) in the Himalaya, C30 is clearly the preferred dataset at high elevations.

We next explored the role of outliers in the accuracy of C-Band and X-Band SRTM heights in the Himalaya. We found 16 C90 outliers ranging in error from −177 to 222 m, 15 C30 from −182 to 220 m and 3X30 from −12 to 29 m (Supplementary Table S3). This indicates that all SRTM data contain outliers that need to be filtered before use for their vertical uncertainties to be close to the SRTM goal. Given the wide range of C-Band outliers, using any SRTM C-Band data “as is” will definitely contain errors much higher that the SRTM goal or the continental SRTM uncertainties^6^,^7^. However, where available, X30 data is a much better option for “as is” use as outlier errors are an order of magnitude less than the C-Band data. Our study, therefore, clearly red flags the use of “as is” C-Band SRTM data that is widespread in the earth sciences^22^,^23^,^24^,^25^,^26^,^27^,^28^,^29^. Our results in the Himalaya corroborates the finding that the SRTM accuracy decreases with elevation^9^,^11^,^13^ as the accuracy in the higher Himalayas was the lowest for all the SRTM data, followed by the foothills and then the Himalayan foreland. This is also corroborated by the plot of outlier-filtered SRTM absolute errors as a function of elevation (Fig. S3) that also shows the increase in the absolute errors with the increase in the elevation of the GPS points for all SRTM datasets. Overall the X30 SRTM was the most accurate followed by C30. The void and outlier-filtered X-Band RMSE for all the three regions of the Himalaya complied with the SRTM goal of RMSE 9.73 m (Table 2). The void and outlier-filtered C30 SRTM complied with the SRTM goal for only the Himalayan foreland whereas the C90 SRTM did not comply with the SRTM goal (Table 2). The accuracy of the re-sampled C30_15 was slightly higher (Table 2) than the C30 data with both the Himalayan foreland and foothills RMSE within the SRTM goal. Hence, re-sampling the C30 data to a higher resolution marginally improves the vertical accuracy of the SRTM data. Therefore, where available, only the X30 may be used “as is” in the Himalaya. Outlier and void error analysis must precede the use of C-Band SRTM data in the Himalaya for accurate use.

The accuracy of the Himalayan SRTM data was also compared to data from Peninsular India (Table 2). Except for the X30 data, the accuracy of Peninsular C90 and C30 datasets was better than the Higher Himalaya and comparable to the Himalayan foreland and the foothills. The X30 accuracy for the Peninsular region was lower than the Himalaya due to the non-uniform distribution of the X-Band data as the southern and central part of the Peninsular regions have sparse X-Band data. Except the C90 dataset, all the other Peninsular RMSE complied with the SRTM mission goal and C30 was the most accurate dataset.

Finally, we explored an effective methodology for SRTM data usage in the Indian Himalaya and Peninsula. The statistical results for the complete dataset indicated a positive mean bias (Table 2) that pointed to a systematic error component in the SRTM data. Therefore, removal of the positive mean bias from the data sets makes the SRTM heights closer to the ICP heights and reduces the height error to only the random errors contained in the standard error of mean. This mean correction applied to the complete dataset reduces the C90, C30, C30_15 and X30 RMSE by 4.46, 3.45, 3.40 and 2.89 m respectively (Table 2). After mean correction, the X30 was still found to be the most accurate with RMSE 5.11 m followed by C30_15 with RMSE 6.48 m. The mean correction was also applied to individual SRTM datasets resulting in shifts in the error distribution (Fig. S4). The RMSE of the mean corrected datasets show maximum improvement in the Higher Himalaya where the C- and X-Band RMSE improved by ~7 m and ~2 m respectively (Fig. 5). The Peninsular dataset also improved on correction with the RMSE of C90 reducing by ~5 m, followed by ~4 m for X30 and ~3 m for C30.

The results of our study clearly indicate that there is considerable and variable uncertainty in SRTM heights and users should only use X-Band data “as is”. An initial assessment, re-sampling and mean bias correction is a quick and an effective way of improving the quality of the Digital Terrain Elevation Data (DTED) before its use and to obtain statistically meaningful insights from SRTM heights.

## Conclusions

1. The GPS ICPs require positions with a minimum precision of 5 places after decimal in degrees for accurate pixel registration of SRTM Digital Terrain Elevation Data. The accuracy of the C90 and C30 DEMs also depends on how the DEMs are aligned and detection and correction of horizontal offset, if any, between the C90 and C30 DEMs is essential. We found that the C-Band 90 m and 30 m DEMs are aligned without any horizontal offset implying that area and length computations using C-Band 90 m and 30 m DEMs would have near identical values^36^.
2. The accuracy of the SRTM data in the Himalaya decreases with increase in elevation for all SRTM Digital Terrain Elevation Data. Consequently, we find that the SRTM heights in the Himalayan foreland are most accurate followed by the foothills. The Higher Himalaya heights have the least accuracy.
3. Overall X-Band SRTM heights appear to be most accurate and should be preferred over C-Band wherever available. In the absence of X30, the recently released C30 should be used. C30 is also preferable because the void-filling is ~7 times more accurate than the v4 C90 data although C30 has more data voids compared to C90.
4. The X30 accuracy in Peninsular India is lower than the Himalaya due to non-uniform, sparsely distributed X-Band ICPs. The height accuracy of SRTM C90 and C30 compares with that in the Himalayan foreland and foothills due to good distribution of the C-Band data in Peninsular India. Both the C30 and X30 in the Peninsular India comply with the SRTM goal. The C90 dataset does not comply with the SRTM goal and is the most inaccurate everywhere.
5. A methodology for effective use of SRTM data may involve re-sampling the original DEM to a higher resolution and removing the mean error bias if any. The “as is” C30 was re-sampled to 15 m resolution and mean corrected to obtain the most accurate C-Band data. All the bands of SRTM data showed a positive mean bias and correcting this significantly improved the accuracy of all the SRTM DEMs. An initial assessment, re-sampling and mean bias correction is a quick and an effective way of improving the quality of the DTED before its use to obtain statistically meaningful insights from SRTM heights.

### Methodology

This study involves obtaining (1) precise position estimates of GPS ICPs (2) C- and X-Band SRTM data followed by (3) registration of ICPs on pixels of SRTM data (4) analysis of the horizontal offset of the C-Band DEM and (5) computation of errors, identification of outliers and statistical analysis.

### Precise position estimates of GPS Independent Check Points (ICPs)

**Figure 1:**
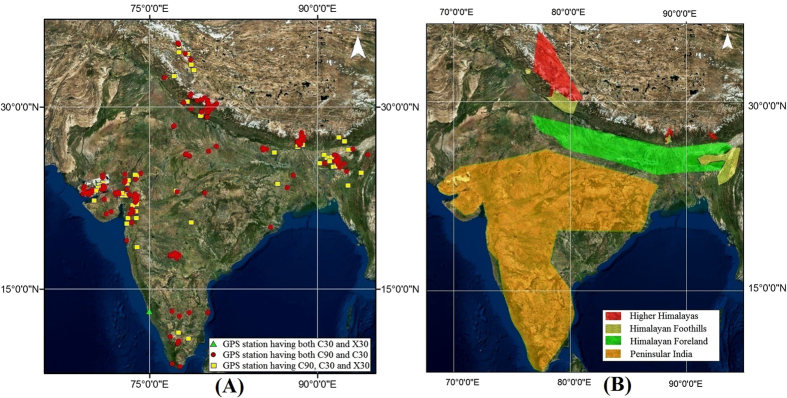
(**a**) Distribution of the Static GPS ICPs used in the study. 156 stations have C90 and C30 SRTM data, 64 stations have all C90, C30 and X30 SRTM data and there is 1 station for which only C30 and X30 SRTM data were available. (**b**) The ICPs were categorized into Higher Himalaya, Himalayan Foothill, Himalayan Foreland, and Peninsular India for analysis. Figure was created using ArcMap 10.1 software from ESRI (Environmental Systems Resource Institute), http://www.esri.com.

**Figure 2:**
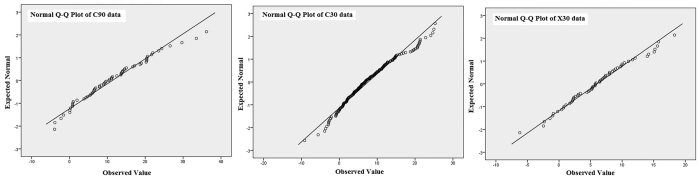
Q-Q Plot for outlier-filtered C90, C30 and X30 errors indicating the non-normal distribution for C-Band errors and an approximately normal distribution for X-Band errors. Figure was created using IBM SPSS Statistics 20 software, http://www-01.ibm.com/software/analytics/spss/products/statistics/.

**Figure 3:**
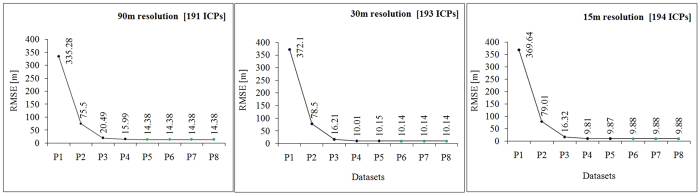
RMSE of complete C90, C30 and C30_15 data for different horizontal precision datasets. The results show that latitude and longitudinal values of minimum precision 5 places after decimal is required to assess the elevation of the 90 m resolution Digital Elevation Models (DEM) and 6 places for both 30 m and 15 m using GPS ICPs. Figure was created using Microsoft Office Excel 2007 software, https://www.microsoft.com/en-in/download/office.aspx.

**Figure 4:**
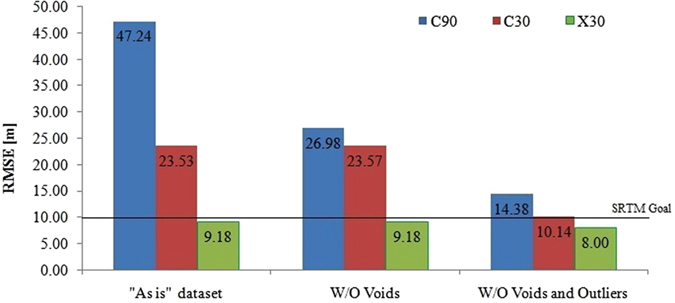
Difference in the accuracy for “as is”, void filtered and outlier filtered C90, C30 and X30 datasets from the Indian sub-continent. Figure was created using Microsoft Office Excel 2007 software, https://www.microsoft.com/en-in/download/office.aspx.

**Figure 5:**
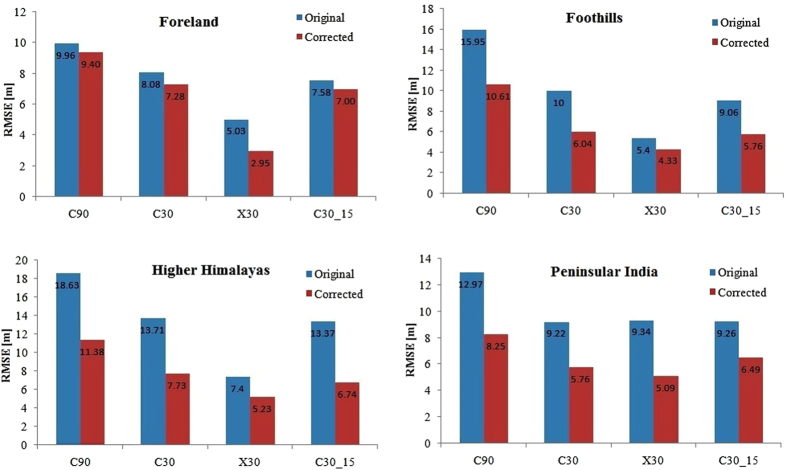
Improvement in the accuracy of the SRTM data after the mean bias correction. Figure was created using Microsoft Office Excel 2007 software, https://www.microsoft.com/en-in/download/office.aspx.

Dual-frequency, differential-static GPS data were collected at the ICPs by making field measurements with a sampling interval of 30 sec. GPS data collected at the ICPs were either (i) 2–3 epochs of data separated by a year with 3 to 5 days in each epoch or (ii) continuous data for 365 days for a minimum period of 1 year. To avoid bias, all the GPS ICPs were located on hard rock (or pillars grouted to them) in regions where GPS signals from satellites were clearly visible and there was minimum vegetation. The data collected at the ICP’s were thoroughly checked for quality using TEQC (Translation, editing and quality check) for high multi-path and cycle slips^37^. Data after the quality check were post-processed along with IGS (International Global Navigation Satellite Systems Service) network data using the GAMIT/GLOBK processing engine which is the global standard in the processing of dual-frequency, high precision static GPS data^38^,^39^. After accounting for errors induced by antenna phase centre variations, satellite and receiver clock bias, orbital accuracy, signal delays due to troposphere and ionosphere, daily positions and uncertainties of ICP’s were generated from loosely constrained network solutions. Precise positions (latitude, longitude and height) of these ICPs were then estimated by combining the daily loose solutions and constraining the IGS sites to their ITRF (International Terrestrial Reference Frame)^40^ positions and associated uncertainties. The post-processed horizontal position of the ICP is the phase-centre position of the GPS antenna with a horizontal precision of 8 and vertical precision of 4 decimal places. The uncertainty associated is of the order of sub-cm to cm in horizontal and vertical positions respectively. The elevations are ellipsoidal heights in the World Geodetic System 1984 datum (WGS1984) with realization based on ITRF2000 (G1150) where 1150 is the GPS week number of ITRF2000 reference frame realization which corresponds to 20 January 2002. The total number of ICPs used to assess the SRTM data were 221 (Fig. 1a; Supplementary Table S1). The entire dataset was next separated into the following categories and analysed (Fig. 1b):

(i) Himalayan foreland (elevation range <300 m) (ii) Himalayan foothills (300–2000 m).

(iii) Higher Himalaya (>2000 m) (iv) Peninsular India.

### Obtaining C90, C30, X30 SRTM Digital Terrain Elevation Data

We used the most recent, void filled^33^, version 4.1^12^ C90 SRTM data as 5 degree × 5 degree tiles in GeoTiff file format from the Consultative Group for International Agriculture Research Consortium for Spatial Information (CGIAR-CSI) website (http://srtm.csi.cgiar.org). The base C90 SRTM data containing voids used to generate the finished versions of SRTM data were also downloaded from the USGS Earth Explorer Interface (http://earthexplorer.usgs.gov/) to study the accuracy of interpolated void heights in the Himalaya. The C30 data were downloaded using the USGS Earth Explorer Interface in the GeoTiff format as 1 degree × 1 degree tiles from http://earthexplorer.usgs.gov/. The orthometric heights from the C90 and C30 tiles were extracted in a 16 bit integer format following the Earth Gravitational Model 1996 (EGM96) geoid vertical datum^2^. The orthometric heights were converted to WGS1984 ellipsoidal heights by adding the geoidal separation for each location. We used the University NAVSTAR Consortium (UNAVCO) Online Geoid Height Calculator to find the geoidal heights at http://www.unavco.org/community_science/sciencesupport/geoid/. This uses the potential coefficient model EGM96 algorithm^41^ with an error range of ±0.5 m to ± 1.0 m^42^. We downloaded the X30 SRTM data from the German Aerospace Center (DLR) from ftp://taurus2.caf.dlr.de/ on the Earth Observation on the WEB (EOWEB) interface as 0.25 × 0.25 degree tiles as WGS84 ellipsoidal heights directly from https://centaurus.caf.dlr.de:8443/eowebng/template/default/welcome/entryPage.vm.

### Registration of ICPs on appropriate pixels of SRTM data

We imported each downloaded SRTM tile as an ArcGIS layer and determined the elevation at the 221 GPS ICPs by registering the ICP locations on the SRTM C90 and C30 pixels “as is”, extracting their corresponding pixel value and converting them to ellipsoidal heights. The C30 tiles were further re-sampled using cubic convolution technique^43^ that determines the value of the pixel by fitting a smooth curve through the 16 nearest input pixel centres to generate 15 m resolution tiles as C30_15. The elevations of these tiles were also extracted for analysis at the 221 ICP positions. The GPS ICP’s were also registered on the 90 m, 30 m and 15 m re-sampled C-Band resolution raster using different precisions of latitude and longitude to explore the minimum horizontal precision required for an ICP for accurate pixel registration. The GPS ICP latitude and longitude used in this study had a precision of 8 places after decimal which allowed us to test this.

### Analysis of the horizontal offset between the C-Band SRTM DEMs

For the vertical error assessment of two DEMs, the alignment of the DEMs is important, particularly in high undulating terrains. The registration error or horizontal offset in a DEM introduces vertical error as the DEM shifts from its true position and planimetric errors are translated in the vertical dimension. The effect of the horizontal offsets is not significant in the plains region but increases with an increase in slope. The relationship between the vertical error and the aspect has been studied previously^13^,^44^,^45^,^46^,^47^. The horizontal offset between the DEMs occur as the data are acquired using different sensors referenced to different datums^46^.

We obtained the C-Band SRTM 90 m and 30 m SRTM DEMs between latitudes 34°15′N and 33°55′N and longitudes 77°40′E and 78°07′E in the Higher Himalaya. Since the DEMs were in different resolution the 30 m SRTM DEM was re-sampled to 90 m SRTM DEM using bi-linear interpolation. The elevation differences raster (Fig. S2a) was obtained by subtracting the pixel values of one DEM from the other. This raster image was compared to the hillshade of the terrain (Figure S2b) obtained using the SRTM DEMs. The hillshade of the terrain is a function based on the slope and aspect and is similar to the elevation difference raster in case the DEMs are unaligned^35^. There was no observed similarity between the hillshade obtained from C-Band DEMs and the elevation difference raster (Fig. S2) indicating the absence of any horizontal offset between the two DEMs. The elevation difference raster had some pixels with high differences ranging between (−151 to 167 m). However ~96% of the elevation difference pixels were within ± 5 m with the mean and SD of 0 m and 3.11 m respectively. The two DEMs were also subjected to multivariate statistical analysis and their covariance and correlation matrix^48^ computed (Supplementary Table S5). The variances were computed by taking the average of the squares of the differences between each cell value of the DEM and the mean value of all the cells. The correlation matrix which is the ratio of the covariance between the two DEMs divided by the product of their SD depicts the dependency between the two datasets. The covariance between the two DEMs was very high at ~400000 m^2^ with a correlation of 0.99999 indicating a near perfect positive relationship between the SRTM 90 m and 30 m DEMs thus further substantiating the absence of any significant horizontal offset.

### Statistical Analysis of SRTM heights

We statistically analyzed “as is” 220, 219 and 65 SRTM C90, C30 and X30 data respectively at the ICPs to quantify and assess SRTM vertical accuracy in the Indian region (Supplementary Table S6, Fig. 1a). We then analysed the data by identifying the SRTM data voids and removing the ICPs located in them and then by also filtering the outliers. The data were then categorized into the Himalayan (foreland, foothills and higher) and the peninsular sets and then analysed with and without the mean correction.

#### Identification of interpolated points falling in the SRTM void

A GIS analysis identified 13 ICPs only in the C-Band data voids in the Himalaya between GPS heights 1327.712 m and 3525.968 m (Fig. S1; Supplementary Table S4). A couple of C30 ICPs in the void region did not have interpolated elevation. The C90 and C30 data from the void regions were separated out and analysed to check on the effectiveness of the void fill algorithm for the SRTM void region (Supplementary Table S4).

#### Analysis and Identification of Outliers from the SRTM datasets

We identified 16 C90, 15 C30 and 3X30 outliers with high errors ranging from −182.20 m to 222.35 m using the Stem-and-Leaf plot (Fig. S5). C90 outlier errors ranged from −177 to 222 m, C30 from −182 to 220 m and X30 from −12 to 29 m. These ICPs were identified and excluded (Supplementary Table S3) from further analysis. The mean GPS elevations of the 16 C90 and 15 C30 outliers were ~1476 m and ~1425 m respectively. The 3 X-Band outliers were from the Himalayan region with mean elevation of ~2206 m.

#### Normality Distribution Tests for Errors

We define the difference between ellipsoidal SRTM and measured heights at an ICP as error. Normality distribution tests (e. g. Shapiro-Wilk’s test^49^,^50^, Q-Q Plots^51^) were done to compute appropriate statistical measures of central tendency, dispersion, symmetry and peakedness for the C90, C30 and X30 error distributions on the void and outlier filtered datasets. In addition, the re-sampled C30_15 errors were also analysed to understand the effect of re-sampling on SRTM accuracy. The statistical analysis involved the computation of the Mean Error, Mean Absolute Error (MAE), Root Mean Square Error (RMSE), Standard Deviation (SD) and Standard Error (SE) for the datasets. The statistical measures were computed with and without the outliers to see the effect of the outlier points on the accuracy.

## Additional Information

**How to cite this article**: Mukul, M. *et al*. Uncertainties in the Shuttle Radar Topography Mission (SRTM) Heights: Insights from the Indian Himalaya and Peninsula. *Sci. Rep.* **7**, 41672; doi: 10.1038/srep41672 (2017).

**Publisher's note:** Springer Nature remains neutral with regard to jurisdictional claims in published maps and institutional affiliations.

## Supplementary Material

Supplementary Data

## Figures and Tables

**Table 1:** Results of the statistical analysis of “as is”, void-filtered and outlier-filtered complete data.

“As is” dataset	*C90*	*C30*	*X30*
# of points	220	219	65
Mean	16.40	8.66	6.48
MAE	21.67	11.71	7.30
SD	44.40	21.93	6.56
SEM	2.99	1.48	0.81
RMSE	47.24	23.53	9.18
**Without voids**	***C90***	***C30***	***X30***
# of points	207	208	65
Mean	10.78	8.30	6.48
MAE	15.39	11.42	7.30
SD	24.79	22.11	6.56
SEM	1.72	1.53	0.81
RMSE	26.98	23.57	9.18
**Without voids and outliers**	***C90***	***C30***	***X30***
# of points	191	193	62
Mean	10.41	7.61	6.15
MAE	11.37	8.11	6.62
SD	9.95	6.71	5.16
SEM	0.72	0.48	0.66
RMSE	14.38	10.14	8.00

**Table 2:** Results of the statistical analysis of errors from void and outlier-filtered and mean-bias corrected data at ICPs in the Indian Himalaya and Peninsula.

Complete dataset (Elevation: −67 m to 4543 m)								
Data	C90	C90 mean corrected	C30	C30 mean corrected	X30	X30 mean corrected	C30_15	C30_15 mean corrected
# of points	191	191	193	193	62	62	194	194
Mean (meters)	10.41	0.00	7.61	0.00	6.15	0.00	7.45	0.00
MAE (meters)	11.37	7.83	8.11	5.35	6.62	4.06	8.02	5.10
SD (meters)	9.95	9.95	6.71	6.71	5.16	5.16	6.50	6.50
SE (meters)	0.72	0.72	0.48	0.48	0.66	0.66	0.47	0.47
RMSE (meters)	14.38	9.92	10.14	6.69	8.00	5.11	9.88	6.48
**Himalayan Foreland (Elevation** **<** **300** **m)**								
# of points	21	21	22	22	7	7	22	22
Mean (meters)	3.27	0	3.5	0	4.08	0	2.9	0
MAE (meters)	7.01	7.15	5.49	5.39	4.14	2.48	5.01	4.96
SD (meters)	9.64	9.64	7.45	7.45	3.18	3.18	7.17	7.17
SE (meters)	2.1	2.1	1.59	1.59	1.2	1.2	1.53	1.53
RMSE (meters)	9.96	9.4	8.08	7.28	5.03	2.95	7.58	7
**Himalayan Foothills (Elevation: 300** **m to 2000** **m)**								
# of points	40	40	39	39	9	9	39	39
Mean (meters)	11.91	0	7.97	0	3.22	0	6.99	0
MAE (meters)	12.26	7.9	8.14	4.91	4.20	3.64	7.83	4.45
SD (meters)	10.74	10.74	6.12	6.12	4.59	4.59	5.84	5.84
SE (meters)	1.7	1.7	0.98	0.98	1.53	1.53	0.93	0.93
RMSE (meters)	15.95	10.61	10.00	6.04	5.40	4.33	9.06	5.76
**Higher Himalaya (Elevation** **>** **2000** **m)**								
# of points	30	30	32	32	13	13	33	33
Mean (meters)	14.75	0	11.33	0	5.23	0	11.54	0
MAE (meters)	16.32	8.89	11.67	6.32	6.41	4.12	11.54	5.72
SD (meters)	11.58	11.58	7.85	7.85	5.45	5.45	6.84	6.84
SE (meters)	2.11	2.11	1.39	1.39	1.51	1.51	1.19	1.19
RMSE (meters)	18.63	11.38	13.71	7.73	7.40	5.23	13.37	6.74
**Peninsular India (Elevation: −67** **m to 2245** **m)**								
# of points	100	100	100	100	32	32	100	100
Mean (meters)	10.01	0	7.2	0	7.83	0	7.29	0
MAE (meters)	10.44	6.7	7.54	4.59	7.98	4.18	7.6	6.09
SD (meters)	8.29	8.29	5.79	5.79	5.17	5.17	5.74	5.74
SE (meters)	0.83	0.83	0.58	0.58	0.91	0.91	0.57	0.57
RMSE (meters)	12.97	8.25	9.22	5.76	9.34	5.09	9.26	6.49
