# Supplementary material for: Uncertainties in the Shuttle Radar Topography Mission (SRTM) Heights: Insights from the Indian Himalaya and Peninsula
Source: Sci Rep. 2017 Feb 8;7:41672. doi: 10.1038/srep41672 (PMC5296860; doi:10.1038/srep41672)
Supplement: Supplementary Data [file srep41672-s1.pdf]

Uncertainties in the Shuttle Radar Topography Mission (SRTM) Heights: Insights from the Indian Himalaya and Peninsula

Manas Mukul[1], Vinee Srivastava[2], Sridevi Jade[3], Malay Mukul[2*]

[1]KIIT University, Bhubaneswar, India 751024; mmukulfca@kiit.ac.in
[2]Dept of Earth Sciences, Indian Institute of Technology Bombay, Mumbai, India 400076; vineesri@iitb.ac.in
[3]CSIR-4PI, CSIR Fourth Paradigm Institute (Formerly CSIR-CMMACS), Wind Tunnel Road, Bangalore, India, 560037; sridevi@csir4pi.in
*Corresponding Author; malaymukul@iitb.ac.in

Supplementary Data

Table S1: Details of all the ICPs used in the study.

Station #	Code	Latitude	Longitude	GPS Elevation (m)	C90 Elevation (m)	C30 Elevation (m)	X30 Elevation (m)
1	AIWL	23.72	92.73	767.993	756.904	766.904	769
2	ALMA	21.58	73.50	199.239	192.151	194.151	X
3	ASKO	29.73	80.28	1268.181	1229.506	1236.506	X
4	AULI	30.53	79.56	2946.778	2928.714	2944.714	X
5	BALA	30.02	80.15	1581.219	1551.276	1564.276	X
6	BBL1	17.96	77.52	519.114	533.052	534.052	X
7	BBL2	17.89	77.62	525.647	521.772	521.772	X
8	BEL	23.90	70.76	182.261	169.752	173.752	173
9	BEL1	30.4	80.17	3553.113	3560.442	3532.442	X
10	BELA	23.89	70.81	-34.587	-35.380	-38.380	-35
11	BERI	26.38	86.14	-9.209	-7.645	-8.645	-9
12	BERJ	30.40	80.16	3496.039	3490.431	3484.431	X
13	BHAG	23.00	72.99	21.646	19.659	21.659	10
14	BHI1	29.34	79.60	1669.747	1671.982	1663.982	1664
15	BHI2	17.65	77.41	568.092	568.899	568.899	X
16	BHO1	23.21	77.45	437.784	436.766	435.766	X
17	BHO2	23.28	77.36	574.780	551.230	555.230	559
18	BHOM	26.62	92.87	11.691	5.181	5.181	11
19	BHOR	22.66	73.82	259.288	236.123	239.123	X
20	BHTW	30.82	78.61	1832.806	1808.081	1826.081	X
21	BHUB	20.26	85.79	-12.961	-19.582	-18.582	X
22	BISH	26.66	93.18	28.226	28.309	27.309	X
23	BMDL	27.27	92.44	2790.110	2776.196	2784.196	2789
24	BOGD	30.22	80.22	2603.696	2646.385	2578.385	X
25	BOKO	26.01	91.29	90.402	89.389	89.389	86
26	BOLA	23.37	69.81	247.311	231.719	236.719	X
27	BOMD	27.27	92.41	2477.837	2456.172	2465.172	2471
28	BULA	26.68	85.44	4.715	8.029	7.029	X
29	BUNI	23.53	87.31	58.269	53.375	54.375	X
30	BURF	30.36	80.19	3525.968	3525.603	3515.639	X

31	CAMH	29.31	80.10	1566.102	1562.205	1560.205	X
32	CHAK	23.15	69.99	74.011	67.754	67.754	66
33	CHAL	29.20	80.08	789.386	822.852	813.852	X
34	CHAM	30.40	78.36	2444.923	2408.746	2425.746	2437
35	CHAU	29.84	80.04	1899.094	1880.675	1884.675	X
36	CHEN	11.16	77.59	439.944	417.957	424.957	430
37	CHIA	30.11	80.83	3455.671	3365.866	3437.826	X
38	CHIT	23.39	70.68	98.383	91.922	92.922	X
39	CHSL	33.58	78.65	4542.688	4544.338	4542.338	X
40	CITA	17.80	76.81	601.414	600.542	598.542	X
41	CPDR	26.12	78.55	216.865	215.644	214.644	X
42	CSOS	25.57	91.86	1551.522	1551.957	1548.957	X
43	DAJE	23.69	70.81	-1.271	-8.147	-8.147	X
44	DAJK	23.69	70.83	-11.719	-23.173	-22.173	X
45	DEHR	30.32	78.05	648.717	639.504	641.504	X
46	DELH	28.48	77.13	231.579	230.409	231.409	X
47	DELO	27.09	88.50	1646.574	1645.710	1638.710	X
48	DEVA	12.63	77.63	939.650	932.079	934.079	X
49	DHAN	23.82	86.44	187.985	187.888	186.888	185
50	DIPA	26.75	85.09	-5.015	6.144	4.144	X
51	DUDH	25.96	90.85	60.896	47.200	55.200	55
52	DUNG	30.50	80.18	3941.143	3909.379	3921.379	X
53	DURG	23.53	87.31	59.214	53.375	55.375	X
54	FARA	22.94	69.52	13.711	0.655	11.655	X
55	GAIG	27.98	88.59	4543.101	4531.907	4531.907	X
56	GANG	23.74	70.50	15.594	-4.646	0.354	0
57	GARB	30.13	80.86	3178.037	3280.416	3160.426	X
58	GAUR	17.91	76.93	584.244	576.019	625.416	X
59	GBSK	27.37	88.57	1965.030	1952.999	1960.999	X
60	GHTU	26.15	91.66	13.140	10.994	10.994	X
61	GHTY	26.14	91.74	88.472	72.996	70.996	X
62	GIDD	26.87	88.30	1435.732	1396.602	1414.602	1406
63	GOHI	22.89	73.07	18.996	11.111	10.111	8
64	GORI	24.17	72.82	808.342	762.292	767.292	X

65	GUNJ	30.16	80.87	3164.632	2939.085	3139.072	X
66	GWAL	26.23	78.17	239.214	210.512	238.512	X
67	HANL	32.78	78.96	4468.562	4463.148	4467.148	4466
68	HASA	23.08	72.08	-13.730	-27.680	-25.680	X
69	HATI	26.18	91.48	181.333	133.085	170.085	X
70	HATR	23.45	69.05	162.902	149.716	150.716	X
71	HNLE	32.78	78.96	4487.178	4473.149	4479.149	4480
72	IAOH	32.78	78.97	4301.008	4266.130	4274.130	4279
73	IMPH	24.75	93.92	762.207	753.822	757.822	760
74	JALA	17.81	77.17	578.963	584.564	584.564	X
75	JBPR	23.13	79.88	346.223	341.771	342.771	X
76	JIPT	30.01	80.74	2317.591	1911.720	2318.799	X
77	JMNR	22.47	70.01	-17.922	-32.318	-29.318	-33
78	JNUC	28.54	77.17	198.336	195.371	195.371	X
79	JOWA	25.47	92.36	1200.986	1190.037	1199.037	X
80	JTBR	23.03	73.67	186.232	181.240	183.240	X
81	JURN	23.36	69.98	136.992	122.428	126.428	123
82	KAGA	22.89	73.65	123.682	109.708	111.708	X
83	KAIN	23.86	72.98	440.979	420.548	424.548	431
84	KAJR	22.89	73.65	120.111	109.707	109.707	X
85	KAK1	21.02	73.80	461.344	452.157	455.157	459
86	KAK2	23.50	70.40	92.257	75.524	77.524	87
87	KAL1	30.22	80.91	3698.797	3732.360	3660.360	X
88	KALI	22.17	73.84	157.970	133.598	144.598	150
89	KANA	29.67	80.26	1753.539	1753.930	1739.930	X
90	KAND	23.56	70.69	88.199	69.538	77.538	74
91	KANM	23.40	70.88	41.552	30.580	36.580	X
92	KANP	26.51	80.23	80.473	72.844	71.844	X
93	KANU	24.56	87.83	-14.861	-11.037	-12.037	X
94	KARD	23.95	72.73	318.978	279.293	286.293	X
95	KARI	22.96	71.97	-29.659	-34.037	-33.037	X
96	KATH	29.28	79.55	490.616	480.074	480.074	484
97	KAYA	23.63	69.13	40.132	41.206	42.206	X
98	KESA	21.76	73.39	404.681	363.579	383.579	X

99	KHAN	22.47	73.80	369.033	353.453	359.453	X
100	KHAR	24.57	70.79	310.400	88.049	90.049	X
101	KHAT	23.18	69.80	294.602	269.123	285.123	X
102	KHED	24.12	73.09	267.397	246.785	260.785	262
103	KHOJ	23.01	69.41	50.885	44.983	44.983	X
104	KHOR	23.03	72.24	-14.477	-21.980	-21.980	X
105	KODI	10.23	77.47	2245.429	2241.009	2246.009	X
106	KOTI	32.32	77.19	2515.199	2488.629	2487.629	2500
107	KRIS	12.95	78.51	757.106	740.319	747.319	X
108	KTML	29.64	79.62	1256.350	1256.402	1252.402	X
109	KUNT	29.60	79.59	1460.595	1451.462	1455.462	X
110	KYON	27.37	88.71	3214.600	3191.155	3186.155	X
111	LACH	27.72	88.56	2622.188	2579.448	2603.448	X
112	LAID	25.50	91.67	1841.147	1835.461	1837.461	1836
113	LAKA	24.53	73.83	735.445	720.435	725.435	729
114	LAMB	30.08	80.79	2524.294	2425.880	2532.889	X
115	LANS	29.85	78.68	1768.262	1752.684	1756.684	X
116	LASP	30.29	80.20	3371.221	3355.085	3347.085	X
117	LAVA	27.07	88.66	1881.848	1870.493	1877.493	1877
118	LBTR	26.09	78.54	225.339	222.701	223.701	X
119	LCHU	27.68	88.73	2904.127	2924.738	2906.738	X
120	LEH1	34.13	77.60	3312.990	3306.307	3306.307	3306
121	LILM	30.15	80.24	2104.893	1917.295	2103.405	X
122	LKNG	34.00	78.41	4274.776	4278.646	4277.646	4281
123	LUCK	26.89	80.94	87.750	57.780	62.780	X
124	LUMA	26.22	94.48	899.413	887.776	891.776	X
125	MABU	24.65	72.78	1630.249	1611.150	1625.150	X
126	MAN1	10.66	78.46	48.393	48.292	49.292	41
127	MAN2	21.36	71.09	189.219	182.551	182.551	X
128	MANG	27.47	88.53	965.934	961.971	960.971	X
129	MART	30.32	80.20	3430.066	3406.883	3424.883	X
130	MAS1	22.32	73.71	295.712	245.901	253.901	X
131	MAS2	26.62	85.13	3.408	0.992	0.992	X
132	MAUP	25.70	91.57	1359.609	1341.856	1344.856	X

133	MAWR	25.20	91.99	305.004	305.775	307.775	X
134	MGLB	34.01	78.30	4135.703	4129.661	4128.661	4130
135	MICA	26.30	78.22	130.426	130.304	134.304	X
136	MIJA	13.06	74.94	121.090	X	111.792	108
137	MILA	30.44	80.15	3548.587	3594.009	3528.009	X
138	MIRR	26.84	88.22	880.997	913.765	894.765	893
139	MLRI	30.69	79.89	3318.988	3299.083	3300.106	X
140	MOPE	25.23	91.44	741.107	740.300	741.300	742
141	MORA	23.42	72.96	86.608	80.306	80.306	X
142	MUMB	19.13	72.92	-3.990	-9.326	-8.326	X
143	MUNG	26.98	88.40	790.644	778.844	780.844	X
144	MUNN	25.41	91.84	1822.939	1817.250	1825.250	X
145	MUNS	30.06	80.20	3527.738	3501.570	3513.570	X
146	MUNT	30.06	80.24	2288.139	2258.578	2272.562	X
147	MUTH	33.19	78.70	4147.473	4138.012	4135.012	4139
148	NABH	30.24	80.98	4294.177	4304.812	4272.812	X
149	NADI	32.25	76.31	1869.781	1848.443	1848.443	X
150	NAMC	27.16	88.32	1427.015	1421.448	1421.448	X
151	NANM	12.93	80.18	-21.125	-40.128	-36.128	X
152	NIMC	27.00	88.68	840.059	806.561	825.561	828
153	NONG	25.90	91.86	512.047	510.957	511.957	X
154	NOST	25.67	91.10	965.531	969.467	966.467	968
155	PALR	22.90	72.52	8.704	-1.685	1.315	0
156	PAN1	29.96	80.60	1327.712	1137.323	X	X
157	PAN2	22.87	73.89	226.186	213.945	217.945	X
158	PANA	34.71	77.58	3238.178	3214.373	3215.373	X
159	PANC	26.19	90.59	5.724	-7.844	1.156	0
160	PARN	20.55	72.95	125.292	95.611	100.611	107
161	PATA	23.56	70.94	35.763	18.057	21.057	X
162	PATH	21.37	73.23	153.053	131.952	138.952	X
163	PBTR	26.12	78.54	222.512	222.664	222.664	X
164	PELI	27.30	88.22	2055.680	2035.921	2043.921	X
165	PEOR	22.92	73.21	32.653	26.920	28.920	X
166	PGRH	29.56	80.21	1550.162	1521.426	1529.426	X

167	PIHT	29.60	80.19	1918.737	1908.005	1905.978	X
168	PILW	20.65	73.39	552.067	530.067	544.067	X
169	PLGT	10.82	76.82	68.697	66.042	64.042	X
170	PLNI	10.43	77.56	243.027	240.858	241.858	X
171	PNMK	34.73	77.57	3149.872	3134.402	3136.402	X
172	PONN	08.18	77.68	-67.472	-71.707	-70.707	X
173	PORR	23.13	71.87	-25.41	-36.376	-35.376	X
174	PUNE	18.56	73.88	494.671	485.050	485.050	493
175	RALG	30.18	80.23	2381.987	2336.281	2369.281	X
176	RAME	23.00	73.13	44.346	36.360	36.360	X
177	RAN1	25.26	91.72	1309.911	1290.943	1299.943	1308
178	RAN3	13.03	76.97	828.267	807.058	817.058	X
179	RANE	18.08	77.23	563.678	550.575	552.575	X
180	RICH	22.70	73.62	106.923	84.029	91.029	X
181	ROHA	23.20	69.27	216.564	208.763	212.763	X
182	RTIR	17.88	76.83	609.012	606.890	607.890	X
183	RTPT	26.29	78.22	146.016	132.310	134.310	X
184	RUND	22.89	72.91	3.339	-1.454	-1.454	-4
185	SACK	21.56	71.51	135.019	121.349	123.228	X
186	SAGB	21.57	73.78	537.435	517.055	523.349	531
187	SAMA	23.19	69.48	142.603	135.599	135.599	X
188	SAMT	23.16	69.51	241.907	227.465	233.465	X
189	SHI1	25.53	91.85	1920.097	1907.825	1910.825	X
190	SHI2	25.57	91.88	1496.580	1482.994	1483.994	X
191	SIDP	22.07	73.48	-8.340	-14.817	-10.817	-6
192	SILC	24.88	92.59	-14.989	-6.378	-11.378	X
193	SISA	24.21	73.72	643.761	631.805	636.805	X
194	SNDN	23.02	68.99	-30.031	-30.911	-31.911	X
195	SOBL	30.05	80.58	1754.467	1661.761	1709.761	X
196	SOLA	23.09	72.52	19.253	14.883	15.883	17
197	SONE	20.63	78.71	224.249	215.240	217.240	219
198	SORE	27.21	88.21	1752.709	1745.477	1745.477	X
199	SUKH	29.17	80.09	1117.226	1106.437	1116.437	X
200	SUKI	31.00	78.68	3505.815	3475.479	3447.549	X

201	SUKP	23.28	70.16	56.798	52.972	54.972	56
202	TANA	24.72	74.19	586.975	553.100	566.100	X
203	TAPO	29.87	80.55	1358.159	1293.450	X	X
204	TARB	21.01	73.06	-19.871	-27.753	-24.753	-22
205	TAWA	27.58	91.94	2443.570	2423.157	2441.157	2445
206	THUL	22.94	72.10	-24.280	-31.204	-30.204	-34
207	TIGE	27.00	88.27	2444.370	2430.714	2438.714	2441
208	TKSH	34.82	77.52	3362.888	3350.617	3350.617	X
209	TNGS	34.03	78.18	4049.672	4036.564	4040.564	X
210	TRTH	34.57	77.62	3121.337	3104.135	3112.135	3111
211	TRVM	08.42	76.97	-17.637	-30.086	-29.086	X
212	TUGN	30.49	79.21	3392.984	3256.625	3379.668	X
213	TURA	25.53	90.21	378.063	377.813	378.813	375
214	TURT	24.34	70.76	105.182	282.378	287.378	X
215	TZPR	26.62	92.78	77.963	66.843	69.843	69
216	UDAI	24.58	73.71	527.685	526.810	526.810	523
217	VANK	22.60	70.93	128.295	128.572	131.572	X
218	VILA	17.99	77.39	570.402	566.191	570.191	X
219	WADR	23.19	72.20	-6.336	-15.448	-14.448	X
220	WIH2	30.33	78.01	622.530	614.202	613.202	X
221	WILM	25.51	90.65	233.274	243.234	231.234	X

Table S2 : RMSE for different precisions after decimal of ICP latitude and longitude for C90, C30 and C30_15m SRTM data.

**90m Resolution**		**30m Resolution**		**15m Resolution**	
Datasets	*RMSE (m)*	*Datasets*	*RMSE (m)*	*Datasets*	*RMSE (m)*
P1	335.28	P1	372.1	P1	369.64
P2	75.50	P2	78.50	P2	79.01
P3	20.49	P3	16.21	P3	16.32
P4	15.99	P4	10.01	P4	9.81
P5	14.38	P5	10.15	P5	9.87
P6	14.38	P6	10.14	P6	9.88
P7	14.38	P7	10.14	P7	9.88
P8	14.38	P8	10.14	P8	9.88

Table S3: Outlier ICPs for SRTM data from Stem-Leaf and Box plot analysis.

**C90 Outliers**							
**S. No**	**Station #**	**Code**	**Latitude**	**Longitude**	**GPS Elevation (m)**	**C-Band Elevation (m)**	**C-Band Error (m)**
1	24	BOGD	30.22	80.22	2603.696	2646.385	-42.690
2	33	CHAL	29.20	80.08	789.386	822.852	-33.466
3	64	GORI	24.17	72.82	808.342	762.292	46.050
4	69	HATI	26.18	91.48	181.333	133.085	48.248
5	87	KAL1	30.22	80.91	3698.797	3732.360	-33.563
6	94	KARD	23.95	72.73	318.978	279.293	39.685
7	98	KESA	21.76	73.39	404.681	363.579	41.102
8	100	KHAR	24.57	70.79	310.400	88.049	222.351
9	111	LACH	27.72	88.56	2622.188	2579.448	42.740
10	119	LCHU	27.68	88.73	2904.127	2924.738	-20.611
11	130	MAS1	22.32	73.71	295.712	245.901	49.811
12	137	MILA	30.44	80.15	3548.587	3594.009	-45.422
13	138	MIRR	26.84	88.22	880.997	913.765	-32.768
14	175	RALG	30.18	80.23	2381.987	2336.281	45.706
15	195	SOBL	30.05	80.58	1754.467	1661.761	92.706
16	214	TURT	24.34	70.76	105.182	282.378	-177.196
**C30 Outliers**							
**S. No**	**Station #**	**Code**	**Latitude**	**Longitude**	**GPS Elevation (m)**	**C-Band Elevation (m)**	**C-Band Error (m)**
1	3	ASKO	29.73	80.28	1268.181	1236.506	31.675
2	6	BBL1	17.96	77.52	519.114	534.052	-14.938
3	33	CHAL	29.20	80.08	789.386	813.852	-24.466
4	58	GAUR	17.91	76.93	584.244	625.416	-41.172
5	64	GORI	24.17	72.82	808.342	767.292	41.050
6	72	IAOH	32.78	78.97	4301.008	4274.130	26.878
7	87	KAL1	30.22	80.91	3698.797	3660.360	38.437
8	94	KARD	23.95	72.73	318.978	286.293	32.685
9	100	KHAR	24.57	70.79	310.400	90.049	220.351
10	106	KOTI	32.32	77.19	2515.199	2487.629	27.570
11	110	KYON	27.37	88.71	3214.600	3186.155	28.445
12	130	MAS1	22.32	73.71	295.712	253.901	41.811
13	138	MIRR	26.84	88.22	880.997	894.765	-13.768
14	195	SOBL	30.05	80.58	1754.467	1709.761	44.706
15	214	TURT	24.34	70.76	105.182	287.378	-182.196

X30 Outliers							
S. No	Station #	Code	Latitude	Longitude	GPS Elevation (m)	X-Band Elevation (m)	X-Band Error (m)
1	62	GIDD	26.87	88.30	1435.732	1406	29.732
2	72	IAOH	32.78	78.97	4301.008	4279	22.008
3	138	MIRR	26.84	88.22	880.997	893	-12.003

Table S4: Details of the ICPs in the SRTM void region in the Himalaya.

S. No	St. #	Code	Latitude	Longitude	GPS Elevation (m)	C90 Error (m)	C30 Error (m)	C30_15 Error (m)
1	30	BURF	30.36	80.19	3525.968	0.36	10.33	2.33
2	37	CHIA	30.11	80.83	3455.671	89.80	17.84	9.85
3	57	GARB	30.13	80.86	3178.037	-102.38	17.61	9.61
4	65	GUNJ	30.16	80.87	3164.632	225.55	25.56	10.56
5	76	JIPT	30.01	80.74	2317.591	405.87	-1.21	2.79
6	114	LAMB	30.08	80.79	2524.294	98.41	-8.60	-3.60
7	121	LILM	30.15	80.24	2104.893	187.60	1.49	0.49
8	139	MLRI	30.69	79.89	3318.988	19.90	18.88	18.88
9	146	MUNT	30.06	80.24	2288.139	29.56	15.58	14.58
10	156	PAN1	29.96	80.60	1327.712	190.39	VOID	VOID
11	200	SUKI	31.00	78.68	3505.815	30.34	58.27	59.27
12	203	TAPO	29.87	80.55	1358.159	64.71	VOID	VOID
13	212	TUGN	30.49	79.21	3392.984	136.36	13.32	9.32
**C90: Mean Absolute Error (MAE)=121.63m and Root Mean Square Error (RMSE)=161.78m**								
**C30: Mean Absolute Error (MAE)=17.15m and Root Mean Square Error (RMSE)=22.64m**								
**C30_15: Mean Absolute Error (MAE)=12.84m and Root Mean Square Error (RMSE)=20.22m**								

Table S5: Covariance and correlation matrix of the C-Band 90m and 30m DEMs.

	Covariance		Correlation	
**DEMs**	**C90**	**C30**	**C90**	**C30**
**C90**	403146.3019	403218.6337	1	0.99999
**C30**	403218.6337	403300.6357	0.99999	1

Table S6: C- and X-Band SRTM data at the Static GPS Independent Control Points in India.

**Data Distribution**	**C90 (Himalaya)**	**C30 (Himalaya)**	**X30 (Himalaya)**
Total ICPs	220 (114)	219 (112)	65 (32)
ICPs with coincident C- and X-Bands data points	64 (32)		
Number of ICPs in the SRTM voids	13(13)	11(11)	0
Number of outlier ICPs	16(10)	15(8)	3(3)
Number of GPS ICPs analysed	**191**(91)	**193**(93)	**62**(29)

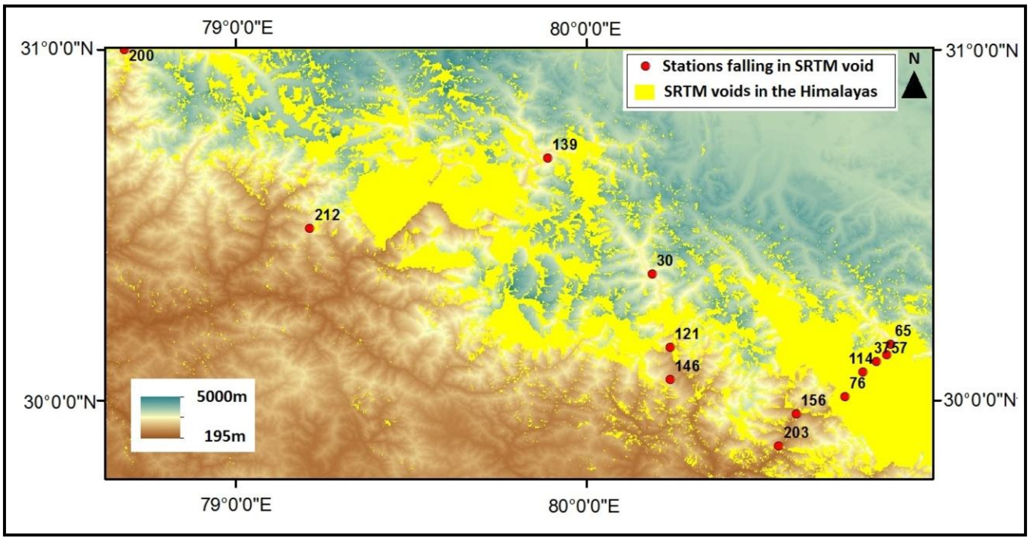

Figure S1: The distribution of GPS ICPs Station in the SRTM data voids in the Himalaya. 13 C-Band data in our study were located in the SRTM void and hence filtered from the analysis. No X-Band data were in the SRTM void region. GPS ICP Station numbers follow Supplementary Table S4. The figure was created using ArcGIS (ArcMap 10.1) software from ESRI (Environmental Systems Resource Institute), http://www.esri.com.

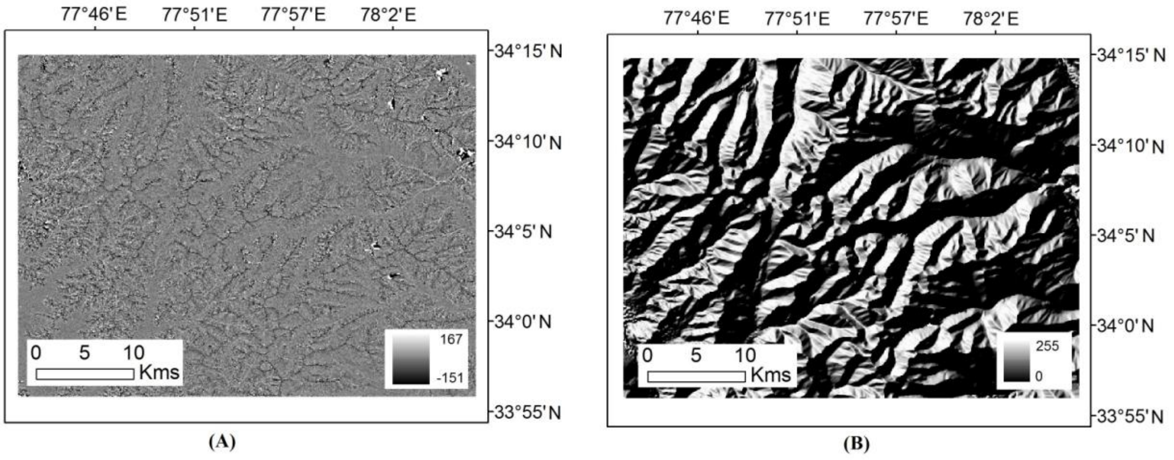

Figure S2: The elevation difference raster (a) between SRTM C-Band 90m and 30m DEMs showing no remarkable similarity to the hillshade of the DEMs (b). The hillshade of both the DEMs were similar and hence only one is shown. The figure was created using ArcGIS (ArcMap 10.1) software from ESRI (Environmental Systems Resource Institute), http://www.esri.com.

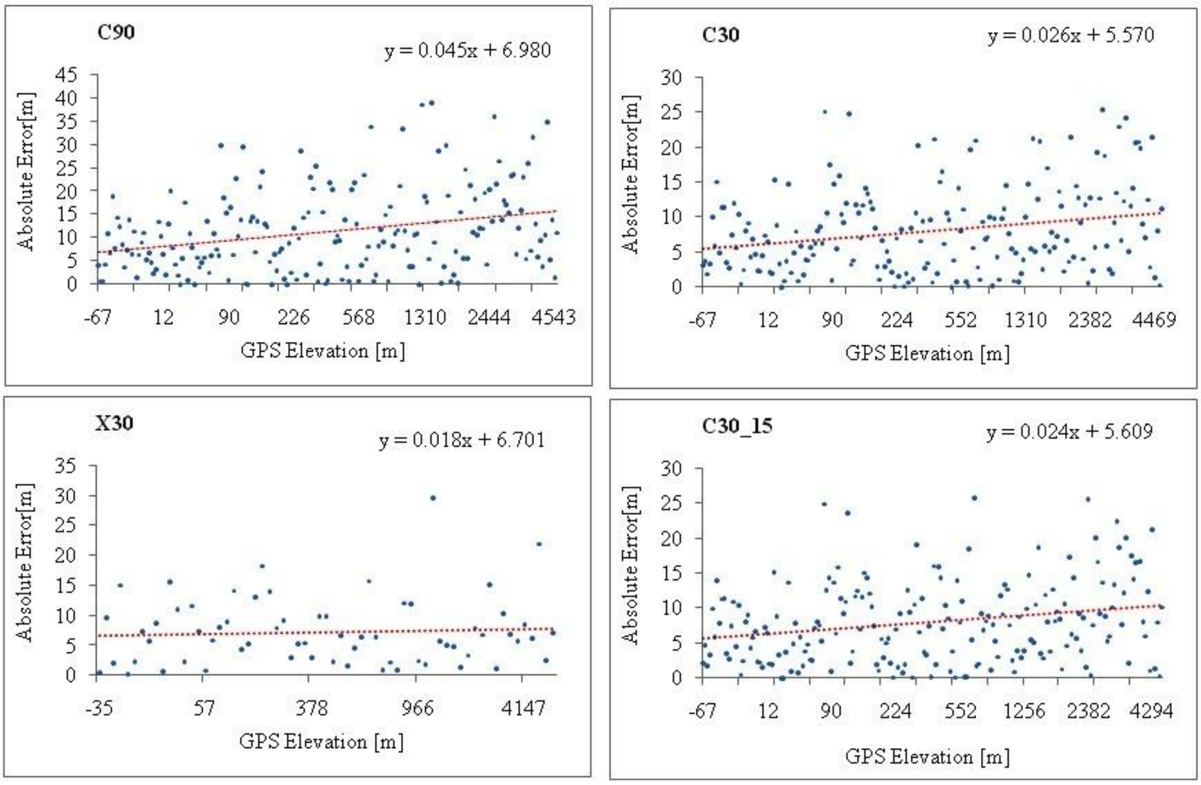

Figure S3: The plot of outlier filtered absolute errors as a function of GPS elevation. The positive slope of the linear trend line for all the datasets indicate the increase in error with the increase in the elevation. The figure was created using Microsoft Office Excel 2007 software, https://www.microsoft.com/en-in/download/office.aspx.

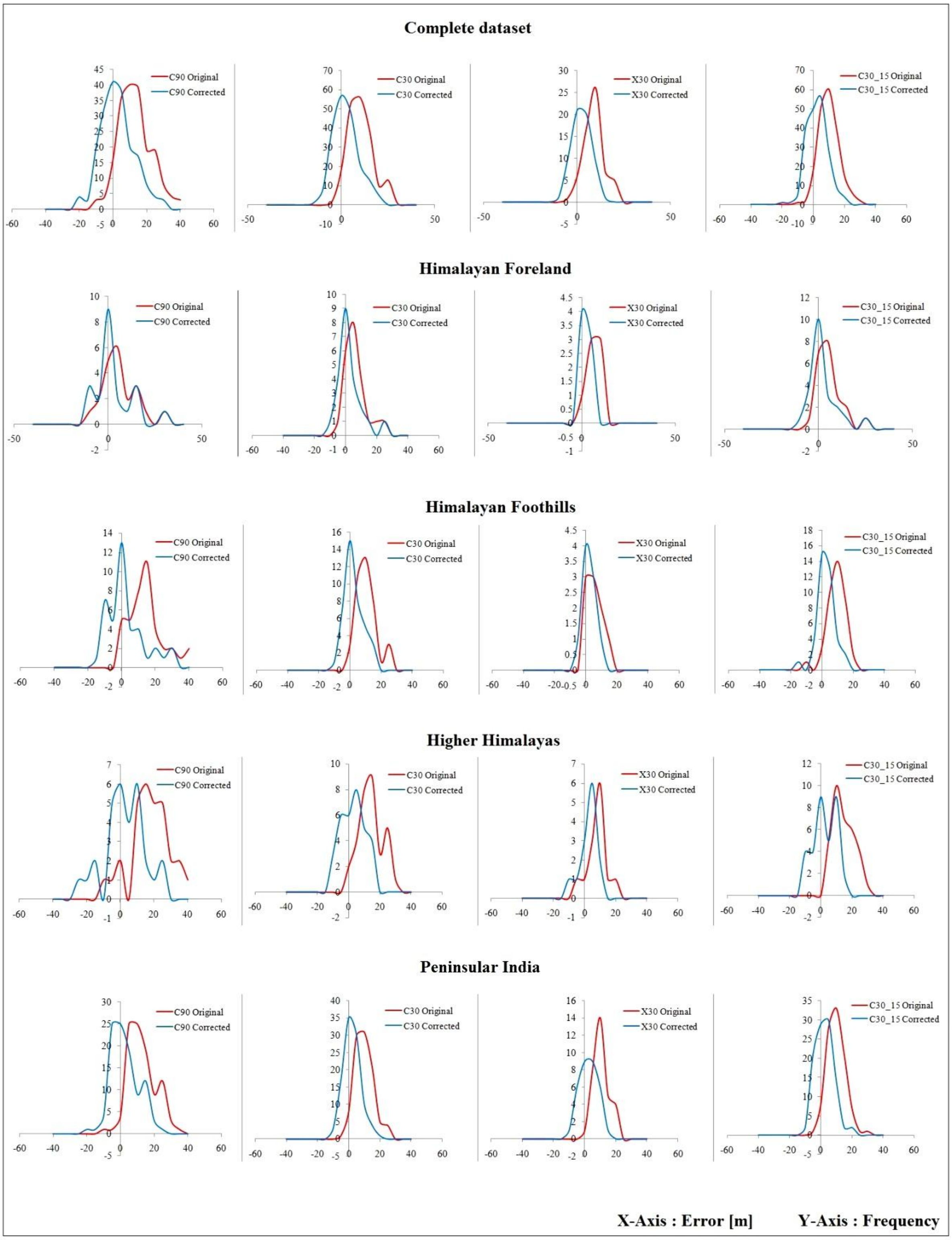

Figure S4:The shift in the distribution curve for C90, C30, X30 and C30_15 data using computed mean error (mean error correction) of the void and outlier filtered ICP data. The figure was created using Microsoft Office Excel 2007 software, https://www.microsoft.com/en-in/download/office.aspx.

C90

```
Frequency    Stem &  Leaf

  7.00 Extremes    (=<-21)
  3.00       -1 .  013
  4.00       -0 .  5789
 16.00       -0 .  0000000011123333
 36.00        0 .  000000000011111122222223333444444444
 40.00        0 .  5555555556666666666677777777778889999999
 39.00        1 .  000000001111111222222333333333333334444
 19.00        1 .  5555556667778888999
 19.00        2 .  0000011111223333344
  8.00        2 .  56688999
  4.00        3 .  1334
  3.00        3 .  689
  9.00 Extremes    (>=40)

 Stem width:     10.00
 Each leaf:       1 case(s)
```

C30

```
Frequency    Stem &  Leaf

  5.00 Extremes    (=<-14)
  1.00       -0 .  9
   .00       -0 .
  1.00       -0 .  5
 10.00       -0 .  2222222333
  9.00       -0 .  000000000
 18.00        0 .  000000001111111111
 25.00        0 .  2222222222222333333333333
 25.00        0 .  4444444444555555555555555
 20.00        0 .  66666666667777777777
 21.00        0 .  888888888999999999999
 20.00        1 .  00000001111111111111
 10.00        1 .  2222222333
 12.00        1 .  444444444555
  3.00        1 .  667
  4.00        1 .  8999
  9.00        2 .  000001111
  1.00        2 .  2
  4.00        2 .  4445
  1.00        2 .  6
 10.00 Extremes    (>=27)

 Stem width:     10.00
 Each leaf:       1 case(s)
```

X30

```
Frequency    Stem &  Leaf
  1.00 Extremes    (=<-12)
  1.00       -0 .  6
   .00       -0 .
  2.00       -0 .  22
  4.00       -0 .  0011
  6.00        0 .  000111
  8.00        0 .  22222233
 11.00        0 .  44455555555
 10.00        0 .  6666677777
  8.00        0 .  88889999
  3.00        1 .  011
  3.00        1 .  233
  5.00        1 .  45555
   .00        1 .
  1.00        1 .  8
  2.00 Extremes    (>=22)

 Stem width:     10.00
 Each leaf:       1 case(s)
```

Figure S5: Stem-and-Leaf plot for C90, C30 and X30 SRTM data showing outliers. The figure was created using IBM SPSS Statistics 20 software, http://www-01.ibm.com/software/analytics/spss/products/statistics/
